# Epidemiology of Acute Pancreatitis in the North Adriatic Region of Croatia during the Last Ten Years

**DOI:** 10.1155/2013/956149

**Published:** 2013-02-14

**Authors:** Davor Stimac, Ivana Mikolasevic, Irena Krznaric-Zrnic, Mladen Radic, Sandra Milic

**Affiliations:** Division of Internal Medicine, Department of Gastroenterology, University Hospital Rijeka, Rijeka, Croatia

## Abstract

*Introduction*. Several European studies have reported an increase in the incidence rate of acute pancreatitis (AP). Therefore, we studied the incidence rate of AP in the North Adriatic Region in Croatia, as well as epidemiological analysis concerning etiology, age, gender, and severity of disease. *Methods*. We analyzed 922 patients with confirmed diagnosis of AP (history, clinical and laboratory findings, and imaging methods) admitted to our hospital during a ten-year period (2000–2009). Epidemiological analysis was carried out focusing on incidence, demographic data, and etiology, as well as severity of the disease based on the Ranson and APACHE II scores. *Results*. The incidence rate varied from 24 to 35/100 000 inhabitants annually. Mean age was 60 ± 16 years. There were 53% men and 47% women among the patients. Most frequent etiologies of AP were biliary stones in 60% and alcohol abuse in 19% of patients. According to the Ranson and APACHE II scores, pancreatitis was considered to be severe in 50% and 43% of the cases, respectively. *Conclusion*. In our region the incidence of AP was around 30 per 100,000 population per year during the ten-year period studied. The mean age at admission was 60 years and etiology was predominantly biliary. In our region, we have shown epidemiological characteristics of AP typical for Mediterranean countries.

## 1. Introduction

Acute pancreatitis (AP) represents an inflammatory disorder of the pancreas [1]. Due to the possibility of local and systemic complications, these patients are admitted to departments of internal medicine or surgical wards for further monitoring and treatment. The exact place of patient's admission depends on the country's tradition, or/and institutional work organization. Knowledge of the disease etiology is important, as early treatment can prevent local and systemic complications [2].

Incidence rate of AP varies in different parts of the world and the actual figures are mainly based on retrospective analyses of hospital admissions. Published studies have shown discrepant results in the incidence rates of AP, ranging from 10 to 80 new cases per 100,000 inhabitants annually [3–9]. There are considerable geographical differences, for example, a low incidence rate in the Netherlands [4] and UK [5] (10 and 24 pts/100,000 inhabitants/year), and a high incidence rate in the Scandinavian countries [6–8] and USA [9] (35 to 73 pts/100,000 inhabitants/year). There are also regional divergences with regard to the precipitating cause. In Finland [8] and USA [10] the main cause of AP is alcohol, whereas studies from Hong Kong [11], England [5], Italy, and Greece [12] showed biliary AP to be more common. 

This is the first published study on the epidemiology of AP in the Croatian population. The aim was to determine the incidence of AP in the North Adriatic Region during a ten-year interval (2000–2009) and to analyze epidemiological factors (demographics, gender, age, and etiology) in patients with AP admitted to our hospital.

## 2. Patients and Methods 

The incidence of AP in the North Adriatic Region of Croatia was calculated according to the 2001 census; there were 305,505 inhabitants (147,215 M/158,290 F) living in the region, and the incidence rate is presented as the number of new cases per 100.000 inhabitants.

The area of the region is 3.577 km^2^ and the average population density is 85 inhabitants per square kilometer. The population is primarily urban, with the inhabitants living in 14 cities. Being the referral center for pancreatic disease and the only hospital in our region, entire population of Northern Adriatic Region gravitate to our hospital. In our institution, patients with acute pancreatitis are hospitalized at the Department of Gastroenterology, Division of Internal Medicine.

All patients admitted to our hospital in the period from January 1, 2000, up to December 31, 2009, with a typical history including the onset of upper abdominal pain (nausea and/or vomiting) within 48 h prior to admission and the elevation of the serum amylase activity at least 3 times greater than the upper limit of normal, were considered to have AP.

Only the patients having the first attack of AP were included in the study. Patients with a relapse of AP or a relapse of chronic pancreatitis were excluded. The diagnosis of AP was additionally confirmed with imaging methods (abdominal ultrasound and/or CT scan), and in some patients hospitalized after year 2003, magnetic resonance (MR), magnetic resonance cholangiopancreatography (MRCP), or endoscopic ultrasound (EUS) were also done.

For the purpose of this epidemiological retrospective study patients were, according to etiology, divided into four groups: alcoholic, biliary, hypertriglyceridemic, and other. Biliary etiology was defined as the presence of gallstones determined by at least one of the imaging methods (abdominal ultrasound, CT, MRCP, or EUS). Alcoholic AP was considered in patients with confirmed alcohol consumption without cholelithiasis/choledocholithiasis, metabolic disorders (hypertriglyceridemia, hypercalcemia), or other possible causes of AP (trauma, drugs, etc.). Hypertriglyceridemia was considered as the cause of AP when the serum triglyceride level was above 11.3 *μ*mol/L. 

The severity of AP was determined by the APACHE II (on admission) and Ranson scores 48 hours upon admission [13]. Severe AP was considered if the APACHE II score was ≥8 and/or Ranson score was ≥3. 

The collected data were formatted in a computer database using Microsoft Access (Microsoft Inc. USA), while statistical and data analysis was performed using statistical software MedCalc, 8th edition. We used *χ* ^2^-test for categorical data analysis and ANOVA for variance analysis. Multiple regression was used to determine independent predictors of severe acute pancreatitis. Incidence rate was calculated on 100.000 residents. *P* value <0.05 was considered to be statistically significant. 

## 3. Results

This epidemiological retrospective study included a total of 922 patients with AP. 

**Figure 1:**
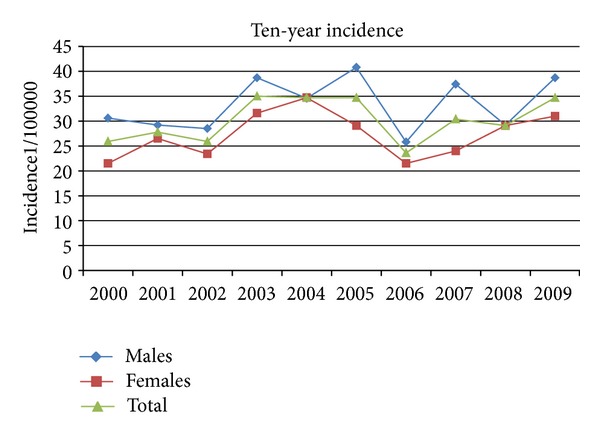
The incidence of acute pancreatitis according to gender during the ten-year period.

There were 53% of men (mean age 59 ± 15) and 47% of a woman (mean age 63 ± 16). Although there is a similar occurrence of the disease between the two sexes in the ten-year period, we found that the first attack of the disease occurs at higher age in women than in men (*P* < 0.001). The incidence of acute pancreatitis in the North Adriatic Region for the period of 2000–2009 is presented in Figure 1. 

**Figure 2:**
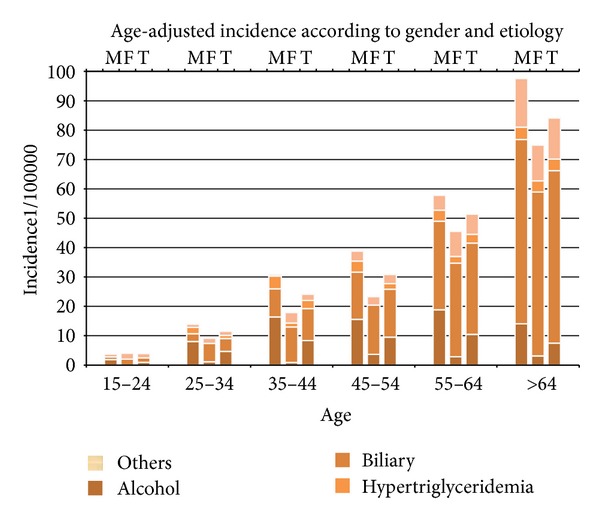
The incidence and etiology of acute pancreatitis change with aging. (M: male; F: female; T: total).

**Figure 3:**
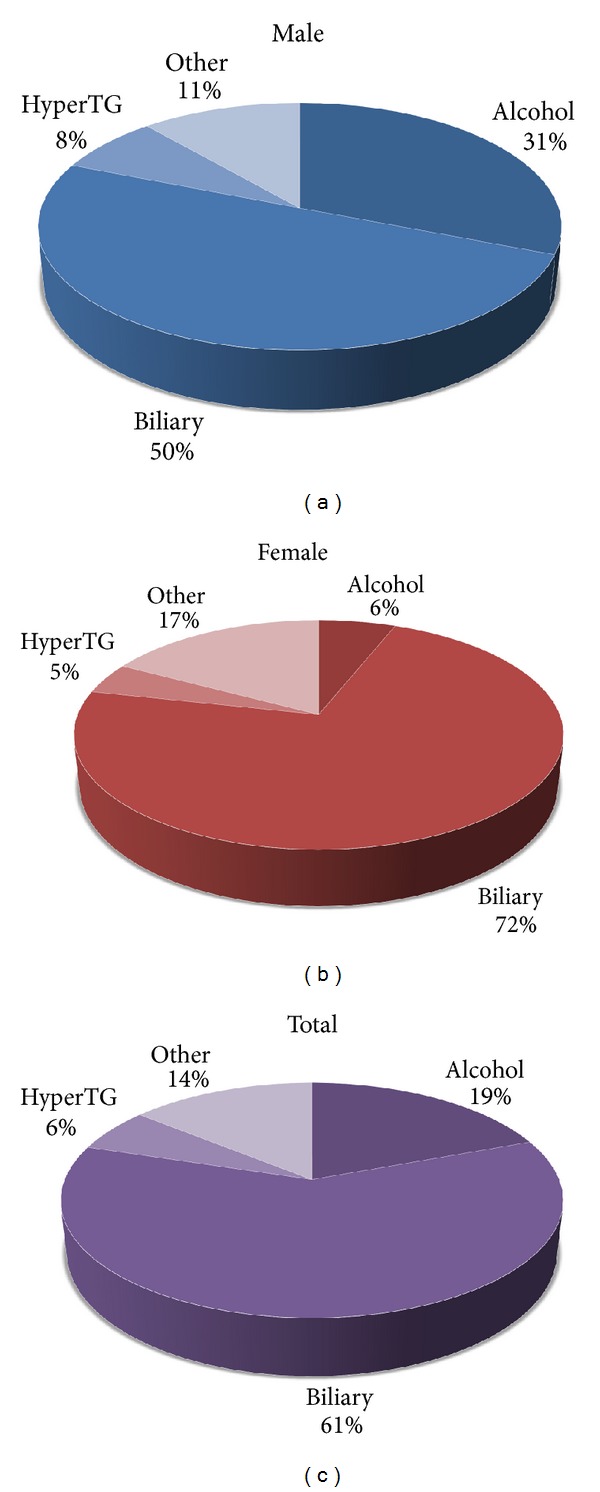
(a) The commonest causes of AP in men (HyperTG: hypertriglyceridemia). (b) The commonest causes of AP in female patients. One should note the rather low proportion of alcoholic AP as opposed to men (HyperTG: hypertriglyceridemia). (c) The commonest causes of AP in all patients (HyperTG: hypertriglyceridemia).

**Figure 4:**
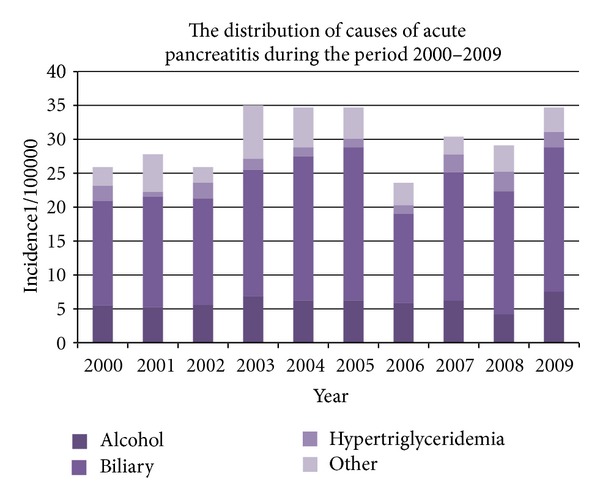
The distribution of the commonest etiologies of acute pancreatitis in the observed period.

The age distribution of the incidence of AP in the ten-year period in the North Adriatic Region is shown in Figure 2. There is an obvious increase in the incidence of AP with age in both sexes. In men, this increase is more pronounced in the forties, while in women the incidence is higher in their fifties and sixties. In the analyzed group, biliary etiology was the most frequent cause of AP. Gallstones were the dominant cause of acute pancreatitis in both genders (Figures 2, 3(a), 3(b), and 3(c)). Although we did not find a significant difference in the occurrence of biliary pancreatitis between men and women, there were a significantly higher proportion of men having alcoholic pancreatitis (*χ* ^2^-test, *χ* ^2^ = 85.122, *P* < 0001). There were no differences in the occurrence of certain etiologies within the specified time period. The ten-year period did not show any significant changes in the trend of respective etiologies (Figures 2 and 4.) The patients with the alcoholic etiology of the disease were in average younger than the other groups of patients (*P* < 0.001). The patients with the biliary etiology represent the oldest group of patients (*P* < 0.001) (Table 1). Thirteen patients had idiopathic pancreatitis.

The severity of pancreatitis was determined according to the Ranson and the APACHE II scores. According to the criterion of Ranson ≥3, 49% of patients had severe pancreatitis and 43% of patients, respectively, had severe AP with APACHE II score ≥8. There was no statistical significance among the various etiologies related to the severity determined by APACHE II and Ranson scores. Regression analysis showed that age was the only demographic factor that determined the severity of disease according to APACHE II and Ranson scores (*P* < 0.001).

## 4. Discussion

The incidence of AP ranges from 10 to 80 new cases/100,000 inhabitants wordwide, but in most European studies it has a much narrower range from 20 to 30 new cases/100,000 [3], that is, in accordance with the results of our study. Although several European studies have shown a significant increase in the incidence over the last 20 years, our study showed a steady trend over the entire ten-year period [2, 5, 14–18]. Our study has also shown an increase in the incidence of AP with regards to the age of the patients. The increase of the incidence with aging can be found in both sexes throughout the study period. The rate of increase differed between the sexes and was more pronounced after the age of 55 in women and after the age of 35 in men. Similar results were posted by the authors of the study conducted in Scotland (1961–1985), with the significant increase in the incidence in younger and middle-aged men (20–59 years) and in elderly women (60 years and older) [16]. In contrast to this findings, Floyd et al. [19] reported a much higher increase in women than in men between 1981 and 2000 (women: 17.1–37.8; men: 18.0–27.1 per 100,000 inhabitants/year). 

In the majority of the studies, gallstones were the predominant etiological factor of AP, except in Denmark (Copenhagen) [19] and Sweden (Goteberg [20] and Stockholm [21]) where alcohol was the predominate cause. Most of the studies have shown that specific etiologic factors dominate within a certain region of the country. For example, a higher proportion of cases with alcoholic AP were observed in the Grampian and Highland region of Scotland [22], compared with other regions in UK [23–26]. The different distribution of afore mentioned etiologies is not entirely clear but can be explained by the difference in alcohol consumption and incidence of cholelithiasis between North and South Europe. Continental Europe has a particularly high incidence of alcoholic pancreatitis, not only because of high alcohol consumption per capita, but also due to the fact that alcoholic beverages in this climate contain a high percentage of alcohol, and as such may have a stronger toxic effect on the pancreas. The frequency of alcoholic and biliary etiology varies throughout the study period, but without significant variances throughout the ten-year period, and as we would expect more women than man had biliary AP and more man than women suffered from alcohol-induced AP. 

In our analysis there were 54 patients (6%) with hypertriglyceridemia as the cause of AP. According to the literature, it is assumed that hypertriglyceridemia is the cause of AP in 4% of cases [2, 27]. The mechanism of disease is not completely understood, but previous studies suggest that damage to pancreatic *α* cells or to the capillary endothelium could be caused by the action of free fatty acids [15]. These data are consistent with other epidemiological studies, especially with the results published in our neighboring country, Italy [12]. Living and eating habits in our county are similar to Italy and other Mediterranean countries and that explains the equal distribution of etiologies and other epidemiological characteristics.

The severity of pancreatitis was determined according to the Ranson score and the APACHE II score. Severe pancreatitis was considered in patients with Ranson score ≥3 and APACHE II score ≥8. Forty-nine percent of patients had severe pancreatitis according to Ranson score and fourty-three percent of patients according to APACHE II score. We found no statistical significance in disease severity graded by Ranson or APACHE II score, according to different etiologies of AP. Multivariate analysis showed age as the only demographic factor that determines the severity of disease according to both scores (*r* = 0.2542, *P* < 0.001), as expected. Although some studies have shown similar results, this phenomenon may be a consequence of a possible bias. Age is actually one of the necessary parameters for calculating the APACHE II and Ranson scores and thus makes a significant part in determining the severity of the disease in these two scoring systems. Also, the serum amylase values are highly dependent on the duration of symptoms prior to admission, and this gives high specificity but not a high sensitivity, so some cases might be missed, especially cases that are admitted late. Thus, some cases of mild pancreatitis are probably missed and this could explain, at least partialy, the high percentage of severe pancreatitis in our study. 

## 5. Conclusion 

In our region the incidence of AP was around 30 per 100,000 population per year during the ten-year period studied. Mean 10-year incidence was 30.2 (CI 95%; 24–36.4). Also, severity of disease was stable. In contrast to several European studies, the number of patients admitted to our hospital due to AP during the last ten years does not fluctuate significantly. Our study has shown that the North Adriatic Region has typical epidemiological characteristics of AP as neighbor Mediterranean countries like Italy and Greece.

## Figures and Tables

**Table 1:** The average age of patients with regards to the etiology of acute pancreatitis.

Etiology	Total		Male		Female	
	*N* (%)	Mean age ± SD	*N*	Mean age ± SD	*N*	Mean age ± SD
Alcohol	178 (19%)	52 ± 15	152 (31%)	50 ± 14	26 (6%)	59 ± 14
Billiary	558 (61%)	63 ± 15	246 (50%)	63 ± 14	312 (72%)	63 ± 16
Hypertriglyceridemia	56 (6%)	57 ± 16	37 (8%)	53 ± 15	19 (4%)	66 ± 15
Other	130 (14%)	61 ± 17	56 (11%)	63 ± 16	74 (18%)	60 ± 17
